# The Long-Term Prognostic Significance of Circulating Tumor Cells in Ovarian Cancer—A Study of the OVCAD Consortium

**DOI:** 10.3390/cancers13112613

**Published:** 2021-05-26

**Authors:** Eva Obermayr, Angelika Reiner, Burkhard Brandt, Elena Ioana Braicu, Alexander Reinthaller, Liselore Loverix, Nicole Concin, Linn Woelber, Sven Mahner, Jalid Sehouli, Ignace Vergote, Robert Zeillinger

**Affiliations:** 1 Department of Obstetrics and Gynecology, Medical University of Vienna, 1090 Vienna, Austria; alexander.reinthaller@meduniwien.ac.at (A.R.); robert.zeillinger@muv.ac.at (R.Z.); 2 Department of Pathology, Klinikum Donaustadt, 1090 Vienna, Austria; a.reiner@a1.net; 3 Institute of Tumor Biology, University Medical Center Hamburg-Eppendorf, 20251 Hamburg, Germany; Burkhard.Brandt@uksh.de; 4 Institute of Clinical Chemistry, University Medical Center Schleswig-Holstein, Campus Kiel, 24105 Kiel, Germany; 5 Department of Gynecology, European Competence Center for Ovarian Cancer, Campus Virchow Klinikum, Charité, Universitätsmedizin Berlin, 13353 Berlin, Germany; elena.braicu@charite.de (E.I.B.); jalid.sehouli@charite.de (J.S.); 6 Division of Gynecological Oncology, Department of Obstetrics and Gynecology, University Hospitals Leuven, Katholieke Universiteit Leuven, 3000 Leuven, Belgium; liselore.loverix@uzleuven.be (L.L.); ignace.vergote@uzleuven.be (I.V.); 7 Department of Obstetrics and Gynecology, Innsbruck Medical University, 6020 Innsbruck, Austria; nicole.concin@i-med.ac.at; 8 Department of Gynecology and Gynecologic Oncology, University Medical Center Hamburg-Eppendorf, 20246 Hamburg, Germany; lwoelber@uke.de (L.W.); Sven.Mahner@med.uni-muenchen.de (S.M.); 9 Department of Obstetrics and Gynecology, University Hospital, LMU Munich, 81377 Munich, Germany

**Keywords:** primary epithelial ovarian cancer, circulating tumor cells, long-term survival

## Abstract

**Simple Summary:**

In ovarian cancer, often diagnosed at an advanced stage and associated with poor overall survival, with just a third of women surviving five years, the controversial prognostic value of circulating tumor cells (CTCs) is still discussed. Currently CTC diagnostics use either molecular approaches or immunofluorescent staining. Our study shows that, given the heterogeneity and extreme scarcity of CTCs, a multifactorial analysis of CTCs is key. Combining both approaches, qPCR and IF, increased sensitivity and may better capture a treatment-related shift in the molecular phenotypes of CTCs. In addition to a long progression-free interval, the absence of CTCs after treatment was an independent predictor of an excellent outcome in patients who had already survived for five years. Thus, a multifactorial CTC approach can identify patients who have elevated risk of recurrence and death and who may require risk-adapted treatment strategies.

**Abstract:**

Introduction: We previously reported the prognostic impact of circulating tumor cells (CTCs) in a multicenter study on minimal residual disease in primary ovarian cancer. With additional follow-up data, we evaluated the combined CTC approach (CTCs^combo^), in particular for the patients who had survived more than five years. Material and Methods: Blood samples taken at baseline and six months after adjuvant treatment (follow-up) were assessed by quantitative PCR (qPCR) measuring PPIC transcripts and immunofluorescent staining (IF). A positive result with either IF or qPCR was classified as CTC^combo^-positive. Further, PPIC was assessed in the primary tumor tissue. Results: The concordance of IF and qPCR was 65% at baseline and 83% after treatment. Results showed that 50.5% of the baseline and 29.5% of the follow-up samples were CTC^combo^-positive. CTCs^combo^ after treatment were associated with increased mortality after adjusting for FIGO stage (HR 2.574, 95% CI: 1.227–5.398, *p* = 0.012), a higher risk of recurrence after adjusting for peritoneal carcinosis (HR 4.068, 95% CI: 1.948–8.498, *p* < 0.001), and increased mortality after five survived years. Discussion: The two-sided analytical approach revealed CTC subpopulations associated with ovarian cancer progression and may illuminate a potential treatment-related shift in molecular phenotypes. That approach can identify patients who have elevated risk of recurrence and death due to ovarian cancer and who may require risk-adapted treatment strategies.

## 1. Introduction

Ovarian cancer is the second most frequent and the deadliest malignancy of the female genital tract. The poor prognosis is largely attributed to late diagnosis of the disease when it has already spread beyond the pelvis and is difficult to treat. In Europe, only every third woman diagnosed with ovarian cancer survives five years [1]. Yet prolonged survival of more than ten years was observed in about 10–20% of patients [2], depending on patient age, stage of disease at primary diagnosis according to the International Federation of Gynecology and Obstetrics (FIGO) staging system, clinical factors such as residual disease after debulking surgery, and genetic signatures such as germline and somatic BRCA mutations [3].

The majority of ovarian-cancer-related deaths are attributed to intra-abdominal metastases, which may have formed through the direct shedding of malignant cells from the primary site into the peritoneal cavity. Thus, it has long been assumed that hematogenous spread plays a minimal role in ovarian cancer metastasis; nonetheless, the presence and prognostic relevance of circulating tumor cells (CTCs) in the blood was shown by our group amongst others (reviewed by [4]), implying that ovarian cancer cells also follow the hematogenous route for metastatic spread [5,6,7].

In an earlier report on the results of a multicenter translational research study investigating predictive markers for the early detection of minimal residual disease in ovarian cancer [8], we demonstrated the presence of CTCs in prospectively collected blood samples in 26.5% of the patients at primary diagnosis and in 7.7% six months after completion of first-line treatment [5]. In parallel to these protein-based analyses using classic immune-fluorescent staining (IF) of CTCs, we followed a molecular-based approach using quantitative PCR (qPCR). In that approach, we analyzed the expression levels of 11 genes, among them the cyclophilin C (PPIC) encoding gene, which had been selected upon a whole transcriptome analysis of patient tumor tissue and control blood samples. We found that among the selected ovarian-cancer-specific transcripts, only the presence of PPIC mRNA in the enriched blood samples was associated with worse overall (OS) and progression-free survival (PFS) [9]. When compared with IF, qPCR is easier to automate, less user-dependent, and able to analyze more targets in general.

Since these previous reports, the observation period of our patient cohort has been prolonged reaching a median follow-up time of more than ten years. Thus, in the present study, we (1) re-evaluated the prognostic significance of CTCs as assessed by qPCR and (2) asked whether a comprehensive CTC approach combining both techniques (IF and qPCR) could generate additional evidence for prognosis estimates in comparison to the single methods. Furthermore, we (3) added data on PPIC protein expression in primary tumor tissue and in CTC samples. Finally, due to the long follow-up time, (4) we evaluated prognostic factors for the likely survival of patients having already survived for five years.

## 2. Results

### 2.1. Patient Characteristics

In the original analysis showing the impact of CTC-related gene markers on the outcome of patients, the median follow-up for still-living patients was 4.3 years (range of 1 to 69 months). At the time of this updated analyses, a further 56 patients have succumbed to their disease. The death rate of the OVCAD study population was 71.2%, with a median follow-up time of 11.1 years (range 4 months to 13.4 years), with 62 patients still alive. Disease recurrence was observed in 172 (80.0%) patients, while 43 patients (20.0%) did not experience recurrence within a median follow-up period of 9.9 (IQR 5.7–12.0) years. Recurrence was not significantly associated with the histological type/grade of the disease (chi^2^ test *p* = 0.072).

### 2.2. Prognostic Impact of PPIC-Positive CTCs at Baseline

Since our initial report, the number of deceased patients almost doubled from 61 (38%) to 115 (72%) among the 165 PPIC-negative patients and from 15 (47%) to 24 (71%) among the 34 PPIC-positive patients. In contrast, recurrences increased to a smaller extent, from 112 (68%) to just 128 (78%), among the PPIC-negative patients and from 24 (71%) to 29 (85%) among the PPIC-positive patients. In line with our initial report (9), PPIC-positivity at baseline still did not have an impact on OS (median 36 versus 52 months, log-rank *p* = 0.289) or PFS (median 14 versus 20 months, log-rank *p* = 0.119). The clinicopathological characteristics of the patients with baseline blood samples available are shown in [9].

### 2.3. Prognostic Impact of PPIC-Positive CTCs at Follow-Up

Among the 79 PPIC-negative patients by PCR, the number of deceased patients increased from 31 (39%) to 52 (66%) since the initial analysis. All 13 PPIC-positive patients have succumbed to their disease, and the median OS of PPIC-negative patients further increased by more than half a year from 64 months in the previous analysis to 71 months at present (log-rank *p* < 0.001).

Likewise, since the initial analysis, a further 15 of 79 PPIC-negative patients relapsed, resulting in a total number of 65 patients with recurrent disease. The median PFS of PPIC-positive and PPIC-negative patients still differed significantly (11 vs. 21 months, log-rank *p* < 0.001). The clinicopathological characteristics of the patients with follow-up blood samples available are shown in [9].

### 2.4. Concordance of qPCR and IF in CTCs

From the 208 blood samples taken at baseline, both qPCR and IF were performed in 89 cases, while a single sample was assessed by IF only and 110 samples by qPCR only. Likewise, 57 blood samples taken at follow-up were assessed using both methods, 35 samples by qPCR only and eight samples by IF only (see Table S1).

**Figure 1:**
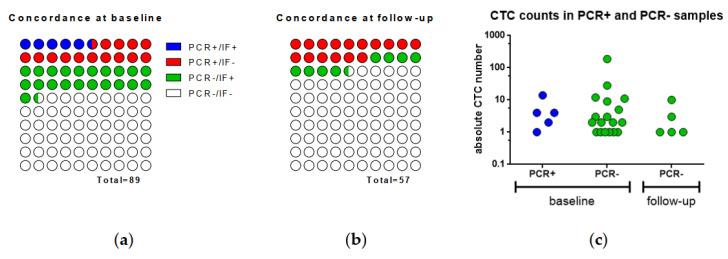
Concordance of PCR and IF in the 89 blood samples taken at baseline (**a**) and in the 57 blood samples taken at follow-up (**b**). The scatterplot (**c**) depicts the CTC numbers assessed by IF at baseline in PCR-positive and PCR-negative samples.

The concordance rate of qPCR and IF at baseline was 65% (Cohen’s κ = 0.009). Thereby, 5 and 52 out of the 89 baseline blood samples were positive or negative by both methods, respectively. Additionally, 13 samples (15%) were assigned as CTC-positive by qPCR only, and 19 (21%) as IF-positive only (Figure 1a). The CTC counts by IF did not differ between the five PCR-positive (median 4 CTCs/mL, range 1–14) and the 19 PCR-negative blood samples at baseline (Figure 1c; median 2 CTCs/mL, range 1–187; Mann–Whitney test *p* = 0.671).

Likewise, in 57 cases, the follow-up blood samples were analyzed using both methods in parallel. Although the CTC counts at follow-up were not statistically different to those observed at baseline according to the Mann–Whitney test (*p* = 0.345), none of the samples was scored as CTC-positive by both methods (Figure 1b). Forty-three (83%) samples were scored as CTC-negative by both methods (concordance 83%, Cohen’s κ = −0.127), whereas nine (17%) were scored as PPIC-positive and five (19%) as IF-positive only.

### 2.5. CTC^combo^ at Baseline and Follow-Up

By adding the positive findings obtained by just one of the methods to the concordant positive results obtained by both methods (CTC^combo^), the overall detection sensitivity increased from 20.2% (18/89 qPCR-positive cases) and 27.0% (24/89 IF-positive cases) to 50.5% (53/105) in the baseline samples. Similarly, in follow-up samples, the detection sensitivity increased from 15.8% (9/57 positive samples by PCR) and 8.8% (5/57 positive samples by IF) to 29.5% (18/61). CTC^combo^-positivity at baseline was associated with the presence of ascites (chi^2^-test *p* = 0.007), while at follow-up, it was more likely in patients with advanced stage at diagnosis (Fisher’s exact test *p* = 0.038; Table 1).

### 2.6. PPIC in the Tumor Tissue

PPIC gene expression in the tumor tissue was not associated with any of the baseline characteristics listed in Table 1, nor with the presence of PPIC-positive CTCs.

Low PPIC protein expression was found in 19 (11.9%) of the cases, medium levels in 63 (39.4%), high levels in 45 (28.1%), and very high levels in 33 (20.6%) of the tumor tissues. However, PPIC gene expression levels were not statistically different between these four groups (see Figure S1). Nevertheless, very high PPIC protein expression (IRS ≥15) was associated with the HGSOC type (chi^2^-test, *p* = 0.034), the presence of peritoneal carcinosis (chi^2^-test, *p* = 0.037), and of PPIC-positive CTCs at follow-up (Fisher’s exact test, *p* = 0.018).

### 2.7. Association of CTC^combo^ and Patient Outcome

**Figure 2:**
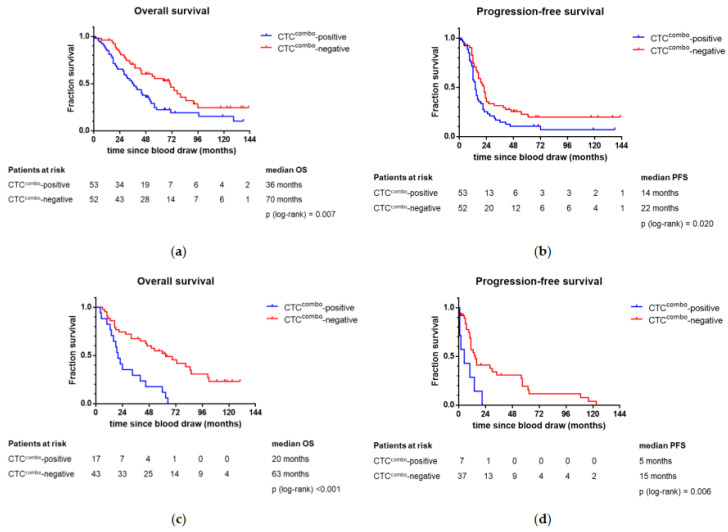
Kaplan–Meier plots showing the association of CTC^combo^ at baseline and follow-up. The patients are stratified into CTC^combo^-positive and CTC^combo^-negative after combining the results from IF and qPCR. The survival differences of CTCs at baseline (**a**,**b**) and at follow-up (**c**,**d**) are evaluated for statistical significance using the log-rank test.

At baseline, CTCs^combo^ occur significantly more often in patients with a short progression-free interval of less than 18 months (chi^2^-test *p* = 0.008) and those who would succumb to their disease within five years after the primary diagnosis (chi^2^-test *p* = 0.005; Table 1). CTC^combo^ at baseline was associated with a higher risk of death and progression in univariate, but not in the multiple analysis (Figure 2a,b; Table S2). 

At follow-up, the presence of CTCs was significantly associated with all parameters indicating worse prognosis, such as poor response assessed at completion of the adjuvant treatment (Fisher’s exact test *p* = 0.007) or six months thereafter (chi^2^-test *p* = 0.001), progression-free interval less than 18 months (chi^2^-test *p* < 0.001), and death within five years after diagnosis (chi^2^-test *p* = 0.012; Table 1). These findings translate into worse OS and PFS in Kaplan–Meier survival analyses (Figure 2c,d) and in both univariate and multiple Cox regression analyses (Table S3). Even after adjusting for the histological type/grade, FIGO stage, and peritoneal carcinosis, CTC^combo^-positivity six months after completion of the adjuvant treatment was independently associated with worse prognosis (OS: adjusted HR 2.574, 95% CI: 1.227–5.398, *p* = 0.012; PFS: adjusted HR 4.068, 95% CI: 1.948–8.498, *p* < 0.001; Table S3).

### 2.8. Prognostic Factors of Long-Term Survival

Seventy-six (35.3%) patients survived for more than five years (LTS), with 27 (12.6%) of them being still alive ten years after the primary diagnosis. The median PFS was significantly longer in the LTS group as compared to the 139 non-LTS patients (39 vs. 14 months; log-rank *p* < 0.001). LTS was significantly associated with lower age, less advanced disease, response to adjuvant treatment, and to the absence of CTC^combo^ at any time (Table 2).

In order to identify prognostic factors for extended long-term survival, we performed a landmark analysis including only those patients who were still alive five years after diagnosis. To this aim, we used a stratified univariate and multivariable Cox regression to allow for the histological type (HGSOC versus other types) with nonproportional hazards affecting OS. Less advanced disease at diagnosis, the absence of peritoneal carcinosis, a progression-free interval of at least 18 months, and the absence of CTCs six months after completion of the adjuvant treatment were significantly associated with an excellent prognosis in these patients (Table 3). Even in the multivariable analysis, the absence of CTC^combo^ at follow-up and a long progression-free interval were independent predictors of excellent long-term survival (Table 3).

## 3. Discussion

CTCs are typically detected either by their specific protein expression or by their nucleic acid content. Here we demonstrate for the first time the complementary information provided by different methodological approaches, such as IF staining of the target cells and mRNA gene expression analysis by qPCR. By pooling positive findings from both techniques, the overall number of positive samples more than doubled compared to each single approach. CTC^combo^-positivity six months after completion of the adjuvant treatment remained independently associated with worse prognosis after adjusting for the histological type and grade, both strongly related with outcome. The absence of CTC^combo^ at the previous follow-up examination six months after completion of chemotherapy was an independent predictor of long-term survival in ovarian cancer patients who had already survived five years after the initial diagnosis.

Given their heterogeneity and scarcity [10], our study yet again points to the importance of a multifactorial analysis of CTCs. In qPCR, we used 11 gene transcripts (PPIC, EpCAM, HER2, and others) for the molecular detection of CTCs [9], while the presence of EpCAM, CK, MUC1, HER2, and EGFR identified CTCs through IF staining [5]. Concordant positive findings were observed in just a few samples, probably due to methodological differences, differences of assay targets and the possible heterogeneity of the CTCs. Of note, concordant results were not more likely in samples with higher CTC numbers. Furthermore, no concordant positive findings at all occurred after treatment, although the CTC counts were not statistically different from those observed at baseline. Thus, qPCR and IF may indeed detect different populations of CTCs. However, the limited number of samples unfortunately does not allow conclusions to be drawn about a potential shift of molecular phenotypes in CTCs provoked by platinum-based adjuvant chemotherapy [11]. Mostert et al. drew similar conclusions from the presence of CTC-specific transcripts in about 50% of IF-negative metastatic colorectal cancer patients [12]. The authors mention another aspect that may be the reason for discordant results in their study as well as in ours, namely the low number of CTCs and the associated stochastic variations due to the Poisson distribution of rare events [13].

The discrepancy between qPCR and IF findings in our study is consistent with observations by others who have compared these technologies for the detection of CTCs in blood or bone marrow. High concordance rates along with low κ values are also reported by others [14,15], mainly because in these studies, as in ours, the majority of the patients are CTC-negative by both methods. For example, discordant results were shown by Strati et al., who compared different technologies for PCR-based detection [16] and Van der Auwera et al., who demonstrated the superior sensitivity of the multimarker quantitative RT-PCR assay compared with the CellSearch System and the AdnaTest for the detection of CTCs in metastatic breast cancer patients [17]. Of note, Strati et al. even reported just moderate κ values when the same primer/probe set was used in singleplex or multiplex RT-qPCR, and no agreement when entirely different primers were used for the same target [16]. In prostate cancer patients, Markou and colleagues demonstrated the advantages of qPCR- over cell-based assays and the characterization of CTCs, not only in terms of sensitivity but also in its openness to performing high-throughput and multiplex analyses [18].

We already reported the considerable variability of CK and EpCAM expression in an individual CTC sample characterized by the large number of 187 CTCs per ml of blood [5,19]. The majority of CTCs were CK-positive, and only 10% were EpCAM-positive [5]; in addition, PPIC-positive CTCs were observed by IF but not by qPCR (data not shown). Low transcript levels were indeed detected, but that sample was deemed CTC-negative because none of the transcripts was above the calculated threshold value. Bearing illegitimate transcription in leukocytes in mind, it is important to emphasize that the number of residual blood cells is a very critical factor in the qPCR-based detection of CTCs. In this study, blood samples were processed using a density gradient enrichment characterized by a poor enrichment factor.

In recent years, a plethora of further label-free technologies have been developed, such as size-based filtration, dielelectrophoretic field-flow fractionation, microvortices, and devices based on deterministic lateral displacement, inertial focusing, or acoustic wave separation (reviewed by [20]). In contrast to label-based methods depending on the expression of specific proteins on the CTCs (positive selection) or hematopoetic cells (negative selection), these label-free methods are based on the premise that CTCs have distinct physical properties, such as larger cell size, smaller density, and increased rigidity compared to blood cells. A further advantage of label-free methods is the copurification of CTC populations that have undergone epithelial to mesenchymal transition and that no longer express typical epithelial cell markers. For example, we used the immunomagnetic CellSearch-based approach for the positive selection of CTCs in the OVCAD study samples; however, we obtained very few positive cases (5/139 baseline and 0/56 follow-up samples) [5]. Thus, the CellSearch-based detection of CTCs was replaced by the multimarker IF-based approach in the remaining set of samples, and this is the main reason for the relatively low number of samples assessed by the CTC^combo^ approach in in the present study.

Molecular CTC profiling by PCR has unveiled myriad biomarkers of potential diagnostic relevance (among them the PPIC gene). Being aware of the advantages of label-free technologies in this regard, our subsequent studies suggested that the microfluidic enrichment of CTCs could be more appropriate for CTC gene expression analyses than density gradient centrifugation [21,22]. The combination of microfluidic enrichment and gene-expression analysis could also be applied for the expanding field of precision oncotherapy in order to select the most promising therapeutic strategy for individual patients based upon the gene expression profile of isolated CTCs [23].

So far, only sparse information exists on PPIC in the context of cancer. PPIC belongs to the cyclophilins, a protein family with peptidyl-prolyl cis–trans isomerase and molecular *chaperone* activities. Among them, cyclophilin A is overexpressed in various cancer types and can protect cancer cells against cellular stress induced by cisplatinum [24]. The prognostic potential of PPIC was shown in high-grade glioma [25] and in ovarian cancer PPIC was co-expressed with a gene signature associated with periostin, a gene product with reported roles in metastasis and angiogenesis [26]. In our earlier study identifying novel markers for the qPCR-based detection of CTCs, we investigated the whole genome expression of a series of gynecologic cancer cell lines and peripheral blood mononuclear cell (PBMC) fractions of healthy donors [27]. Being among the highest differentially expressed genes, PPIC was further evaluated in the blood of cancer patients as a marker for CTCs. Then in the OVCAD study, the gene-expression profile was assessed in paired ovarian-cancer tumor tissue and PBMCs, and again PPIC was one of the most upregulated genes [9]. Of note, the presence of PPIC transcripts was observed in about 70% of the PCR-positive blood samples, and only PPIC was associated with outcome [9]. In our study, a high PPIC protein expression in the primary tumor was related with peritoneal carcinosis, histological type/grade, and PPIC-positive CTCs at follow-up but not at baseline. One explanation for these findings is that PPIC in the tumor tissue may be related with a more aggressive and less chemosensitive phenotype that is more prone to shed CTCs after treatment; additionally, as suggested above, the CTC population may be enriched with PPIC-positive cells after treatment.

Our study is characterized by an exceptionally long median observation period, more than 10 years. The percentage of patients surviving more than five years since their primary diagnosis in our study is broadly in line with the 5-year survival rates reported for European countries [1,28,29]. Given the fact that FIGO I stages were not included in the OVCAD patient cohort, the percentage of patients surviving 10 years or even longer is at least comparable with the 10-year survival rates reported from other studies, which generally include all stages [3,30,31]. The strong association of a long progression-free interval and long-term survival in our study is in line with a recent report [32]. A second major finding was that all patients alive 10 years after diagnosis were assigned as CTC^combo^-negative six months after the completion of the adjuvant treatment. Moreover, the absence of CTC^combo^ after treatment was an independent predictor of an excellent outcome in patients who had already survived for five years. 

Unfortunately, the small sample size gradually lowers the significance of our study. The reasons are that paired baseline and follow-up samples were not taken from all patients and that that the CTC^combo^ approach was not applicable to all samples, because—as already mentioned above—some samples had been analyzed using a modified protocol with low sensitivity [5]. Furthermore, BRCA status was not assessed in the majority of patients, and thus, we were not able to test the effect of BRCA mutations on long-term survival. However, the controversial impact of BRCA in this regard was discussed, and very recently, Baum et al. suggested that the role of BRCA mutations could only be seen in combination with other factors [33].

## 4. Materials and Methods

### 4.1. Study Design

From January 2006 to December 2008, 276 patients with confirmed primary epithelial ovarian cancer were prospectively enrolled within the multicenter OVCAD study, a 6th Framework Program Project (LSHC-CT-2005-018698) of the European Union. The overall goal of the study was to investigate new predictors for the early detection of minimal residual disease. The study protocol was approved by the local ethics committees of the participating OVCAD partners (EK207/2003, ML2524, HEK190504, EK366, and EK260). Detailed inclusion and exclusion criteria, together with clinical data have already been presented elsewhere [8]. The survival data were updated at the end of 2019. Patients surviving for at least five years after the primary diagnosis were regarded as long-term survivors (LTS) according to the definition of Hoppenot et al. [3].

### 4.2. CTC Analysis

Blood samples were taken in six 9 mL-Vacuette EDTA coated blood collection tubes (Greiner Bio-One) after having obtained written informed consent before primary surgery or neoadjuvant chemotherapy (i.e., baseline samples), and six months ± 21 days after completion of the adjuvant chemotherapy (i.e., follow-up samples). All samples had been processed on the same day, using a two-layer density gradient centrifugation as described previously [34] to obtain a monocyte blood fraction possibly containing CTCs. From 215 patients a sample from at least one time-point was available providing sufficient RNA quantity and quality for subsequent gene expression analysis using qPCR [9]. Hence, qPCR was done in 199 baseline and in 92 follow-up samples. In parallel, immunofluorescent staining (IF) was performed in 90 baseline and in 65 follow-up samples. Here, CTCs in the same density gradient-enriched blood fractions were identified due to the presence of epithelial cell adhesion molecule (EpCAM), cytokeratins 7 and 18 (CK7/18), mucin 1 (MUC1), human epidermal growth factor receptor 2 (HER2), and epidermal growth factor receptor (EGFR) [5]. A full outline of the retrospective analyses in baseline and follow-up samples is given in Table S1.

### 4.3. A Complementary CTC Approach by Combining IF and qPCR Results

To investigate whether a combination of both approaches—qPCR [9] and IF [5]—would increase the detection sensitivity of CTCs, we assigned all patients who were positive by either qPCR and IF or both to the CTC^combo^-positive group, whereas patients who were CTC-negative by both methods were assigned to the CTC^combo^-negative group. Blood samples, which were CTC-negative by just one method but had not been processed using the other one due to technical reasons, were omitted, because the negative result had not been confirmed by both approaches.

### 4.4. PPIC Protein Expression in the Tumor Tissue

PPIC protein expression was assessed on tissue microarrays (TMA) created from formalin-fixed paraffin-embedded primary tumor tissue samples as described [35]. Immunohistochemistry was performed after antigen retrieval by microwaving the slides for 15 min in EDTA (1 mM, pH 8.0) and Dako Target Retrieval Solution (Dako, Glostrup Kommune, Denmark). Slides were then cooled to room temperature followed by blocking endogenous peroxidase using a blocking solution (Ultra V Block; TA-015HP, ThermoFisher Scientific, Waltham, MA, USA). The primary polyclonal rabbit antibody against PPIC (1:200; Atlas Antibodies, Cat# HPA039163, AB_2676374) was applied overnight at 4 °C. This was followed by Dako LSAB System (Dako), used according to the manufacturers’ instructions. Diamino-benzidine (DAB, 1:50 in DAB Substrate Buffer, K0673, Dako) was used as a chromogen. Counterstaining was performed using hematoxylin. 

PPIC expression levels were scored semiquantitatively based on staining intensity and the percentage of positive tumor cells and combined with the immunoreactive score (IRS) as described [36,37]: IRS = SI (staining intensity) × PP (percentage of positive cells). SI was determined to be 0 = negative; 1 = weak; 2 = moderate, and 3 = strong. PP was defined as 0; 1, <10%; 2, 10–25%; 3, >25–50%; 4, >50–75%; 5, >75–99%; 6 = 100% positive cells. The patients were stratified by IRS into four groups: PPIC expression absent to low (IRS ≤ 4), medium (IRS 5 to 10), high (IRS 12), and very high (IRS ≥ 15). 

### 4.5. PPIC Gene Expression in the Tumor Tissue

Total RNA was extracted from fresh frozen tumor tissue and further transcribed into cDNA as described [35]. Briefly, RNA was isolated using the ABI PRISM 6100 Nucleic Acid PrepStation (Tissue RNA isolation, Applied Biosystems, Life Technologies, Carlsbad, CA, USA) and quantified spectrophotometrically. cDNA synthesis was performed with 500 ng high-quality RNA (RIN > 5) using the Omniscript Reverse Transcription Kit (QIAGEN) according to the manufacturer’s instructions. qPCR was performed in duplicates with the ViiA7 Real Time PCR System (Applied Biosystems, SCR_019582) using the PPIC TaqMan Gene Expression Assay (Hs00181460_m1, Applied Biosystems, Life Technologies), according to the manufacturer’s instructions. As a reference, the gene expression of the housekeeping gene GAPDH (Hs99999905_m1, Applied Biosystems, Life Technologies) was measured. Two μL cDNA, 0.4 μL TaqMan Gene Expression Assay, 4 μL 2x TaqMan Gene Expression MasterMix (Applied Biosystems, Life Technologies), and 1.6 μL H_2_O were used. The reaction mixture was preincubated at 50 °C for two minutes and at 95 °C for ten minutes, followed by 40 cycles of two-step incubation at 95 °C for 15 s and at 60 °C for one minute. Only samples with GAPDH Ct-values ≤ 26 indicating sufficient RNA quantity and quality were included. Replicate Ct-values showing a standard deviation of >0.5 indicating poor quality of the quantitative value were excluded. The ddCt algorithm was used to assess the PPIC gene expression relative to the reference gene and a cell line calibrator sample [38].

### 4.6. Statistics

Pearson’s chi-square and Fisher’s exact test were used to assess the relationship between the presence of PPIC-positive CTCs and CTCs^combo^, respectively, and the clinicopathological characteristics of the patients. CTC counts were compared using the independent samples’ Mann–Whitney Tests. The linear-to-linear association was used to evaluate the correlation of the PPIC protein immunoreactive score (IRS) in the tumor tissue with the clinicopathological characteristics of the patients and PPIC-positive CTCs, respectively. Nonparametric tests (Mann–Whitney U test and Kruskal–Wallis test) were used to assess the association of PPIC gene expression levels with clinicopathological characteristics, PPIC-positive CTCs, and PPIC IRS. The concordance of positive findings by qPCR and IF was calculated by dividing the number of concordant samples with the total number of analyzed samples and by computing Cohen’s κ values [39].

Clinical endpoints were calculated as follows: progression-free survival (PFS) between the time of blood collection (at primary diagnosis and six months after completion of the adjuvant treatment) and first recurrence, or overall survival (OS) between the time of blood collection (as above) and death due to any cause. Patients without a documented date of recurrence were excluded. Patients surviving for at least five years after the primary diagnosis were regarded as long-term survivors (LTS) according to the definition of Hoppenot et al. [3]. Kaplan–Meier survival analyses and log-rank testing were used to compare survival outcomes [40]. Cox proportional hazards regression was used to determine univariate and multiple hazards ratios for PFS and OS [41], stratified by histological type/grade of the primary tumor into high-grade serous ovarian cancer (HGSOC) and low-grade serous ovarian cancer (LGSOC) or other histological types. Covariates were patient age (≥ 55 versus <55), disease stage according to the International Federation of Gynecology and Obstetrics (FIGO; IIa-IIIb versus IIIc and IV), residual disease after surgery (yes versus no), peritoneal carcinosis (yes versus no), ascites (yes versus no), and the CTC^combo^ (positive versus negative, respectively). A landmark analysis at five years was performed to identify prognostic factors for the survival of patient having already survived for five years. Statistical analysis was performed by SPSS version 19.0 (SPSS Inc., Chicago, IL, USA; RRID: SCR_002865). The level of significance was set at *p* < 0.05.

## 5. Conclusions

In conclusion, our results indicate that a multiple approach is needed to detect rare CTCs, which express heterogeneous phenotypes, per se, and additionally, may undergo a treatment-related shift in molecular phenotypes. Such an approach can identify patients who have an elevated risk of recurrence and death and who may require risk-adapted treatment strategies. Our data set the stage for future studies of the longitudinal analysis of CTCs in serial blood samples and the evaluation of the effect of novel treatment strategies on patient outcome.

## 6. Patents

E.O. and R.Z. filed a patent application for using PPIC as a novel tumor marker in ovarian cancer.

## Figures and Tables

**Table 1:** Association of the combined CTC approach with patients’ characteristics. All patients who were positive by qPCR and/or IF were assigned to the CTC^combo^-positive group. Pearson’s chi-square and Fisher’s exact test (*) were used to assess the association of each parameter and CTC^combo^-positivity at baseline and follow-up.

	Baseline Samples			Follow-Up Samples		
	N	CTC^combo^	*p*	N	CTC^combo^	*p*
Total	105	53 (50.5%)		61	18 (29.5%)	
**Patients’ characteristics at baseline**						
Age ≤55 >55	39 66	19 (48.7%) 34 (51.5%)	0.782	23 38	4 (17.4%) 14 (36.8%)	0.106
FIGO stage IIA–IIIB IIIC IV	8 68 29	3 (37.5%) 32 (47.1%) 18 (62.1%)	0.301 *	8 42 11	0 12 (28.6%) 6 (54.5%)	0.038 *
Histotype LGSOC HGSOC other	6 80 15 ^a^	2 (33.3%) 39 (48.8%) 8 (53.3%)	0.754	4 48 6 ^b^	0 13 (27.1%) 3 (50.0%)	0.248
Grade 1–2 3	23 82	11 (47.8%) 42 (51.2%)	0.774	13 48	4 (30.8%) 14 (29.2%)	1.000 *
Residual disease yes no	37 68	20 (56.0%) 33 (48.5%)	0.589	21 40	9 (42.9%) 9 (22.5%)	0.098
Peritoneal carcinosis yes no	75 30	42 (56.0%) 11 (36.7%)	0.073	45 16	16 (35.6%) 2 (12.5%)	0.114 *
Ascites yes no	77 28	45 (58.4%) 8 (28.6%)	0.007	49 12	16 (32.7%) 2 (16.7%)	0.481 *
**Response to adjuvant treatment**						
At completion cCR cPD, cSD, cPR	78 27	37 (47.4%) 16 (59.3%)	0.290	55 6	13 (23.6%) 5 (83.3%)	0.007 *
Six months after completion cCR cPD, cSD, cPR	66 39	30 (45.5%) 23 (59.0%)	0.181	40 21	6 (15.0%) 12 (57.1%)	0.001
Progression-free interval <18 months ≥18 months	58 47	36 (62.1%) 17 (36.2%)	0.008	29 32	15 (51.7%) 3 (9.4%)	<0.001
Long-term survival LTS (OS ≥ 5y) non-LTS	33 72	10 (30.3%) 43 (59.7%)	0.005	25 36	3 (12.0%) 15 (41.7%)	0.012

^a^ Clear cell and mucinous (each *n* = 1); endometrioid (*n* = 4), mixed epithelial (*n* = 3), and undifferentiated histotype (*n* = 6). ^b^ clear cell and mucinous (each *n* = 1); endometrioid (*n* = 2), mixed epithelial (*n* = 2).

**Table 2:** Baseline characteristics of long-term survivors and non-long-term survivors. Pearson’s chi-square and Fisher’s exact test (*) were used to assess the association of each parameter and long-term survival. *p*-values relate to the comparison of patients surviving at least five or ten years with the non-LTS group.

		Non-Long-Term Survivors	Long-Term Survivors			
	N	OS < 5y	OS ≥ 5y	*p*	OS ≥ 10y	*p*
Total Cases	215	139 (64.7%)	76 (35.3%)		27 (12.6%)	
Median PFS (95% CI)		14 Months (12.5–15.5)	39 Months (24.1–53.9)	<0.001	Not Reached	<0.001
**Baseline characteristics**						
Age ≤55 >55	87 128	41 (29.5%) 98 (70.5%)	46 (60.5%) 30 (39.5%)	<0.001	19 (70.4%) 9 (29.6%)	<0.001
FIGO stage IIA–IIIB IIIC IV	24 153 38	10 (7.2%) 96 (69.1%) 33 (23.7%)	14 (18.4%) 57 (75.0%) 5 (6.6%)	0.001	10 (37.0%) 17 (63.0%) 0 (0%)	<0.001 *
Histotype LGSOC HGSOC other	15 163 28	7 (5.3%) 107 (81.1%) 18a (13.6%)	8 (10.8%) 56 (75.7%) 10b (13.5%)	0.352	4 (15.4%) 18 (69.2%) 4c (15.4%)	0.153 *
Peritoneal carcinosis yes no	147 68	105 (75.5%) 34 (24.5%)	42 (55.3%) 34 (44.7%)	0.002	8 (29.6%) 19 (70.4%)	<0.001 *
Ascites yes no	160 55	109 (78.4%) 30 (21.6%)	51 (55.3%) 25 (44.7%)	0.069	13 (48.1%) 14 (51.9%)	0.002
Residual disease yes no	70 145	55 (39.6%) 84 (60.4%)	15 (19.7%) 61 (80.3%)	0.003	3 (11.1%) 24 (88.9%)	0.005
**Response to adjuvant treatment**						
At completion cCR cPD, cSD, cPR	173 42	99 (71.2%) 40 (28.8%)	74 (97.4%) 2 (2.6%)	<0.001	27 (100.0%) 0 (0%)	<0.001
Six months after completion cCR cPD, cSD, cPR	142 73	72 (51.8%) 67 (48.2%)	70 (92.1%) 6 (7.9%)	<0.001	27 (100.0%) 0 (0%)	<0.001
Progression-free interval <18 months ≥18 months	105 110	90 (64.7%) 49 (35.3%)	15 (19.7%) 61 (80.3%)	<0.001	27 (100.0%) 0 (0%)	<0.001
**Laboratory parameters assessed at baseline**						
CA-125 <35 U/mL ≥35 U/mL Not assessed	19 176 20	14 (11.0%) 113 (89.0%)	5 (7.4%) 63 (92.6%)	0.410	2 (7.7%) 24 (92.3%)	1.000 *
HE-4 < median ≥ median not assessed	101 100 14	58 (43.6%) 75 (56.4%)	43 (63.2%) 25 (36.8%)	0.008	21 (80.8%) 5 (19.2%)	0.001
CTC^combo^ positive negative not assessed	52 53 110	43 (81.1%) 29 (18.9%)	10 (30.3%) 23 (69.7%)	0.005	3 (37.5%) 5 (62.5%)	0.488 *
**Laboratory parameters assessed at follow-up**						
CA-125 <35 U/mL ≥35 U/mL Not assessed	62 34 119	24 (46.2%) 28 (53.8%)	38 (86.4%) 6 (13.6%)	<0.001	15 (100.0%) 0 (0%)	<0.001
CTC^combo^ positive negative not assessed	18 43 154	15 (41.7%) 21 (58.3%)	3 (12.0%) 22 (88.0%)	0.012	0 (0%) 5 (100.0%)	0.309 *

^a^ Mucinous (*n* = 1), endometrioid (*n* = 2), mixed epithelial (*n* = 7), and undifferentiated histotype (*n* = 8); ^b^ endometrioid (*n* = 6), clear cell (*n* = 2), mixed epithelial and mucinous (each *n* = 1); ^c^ endometrioid (*n* = 2), mucinous and clear cell (each *n* = 1).

**Table 3:** Prognostic factors for long-term survival. The Cox regression analysis was stratified by the histological type/grade of the ovarian cancers. Covariates were patient age (≥ versus <55), FIGO (IIA and IIIB versus IIIc versus IV), residual disease after surgery (yes versus no), peritoneal carcinosis (yes versus no), progression-free interval (< versus ≥18 months), and the combined CTC approach (CTC^combo^-positive versus -negative) at baseline and follow-up. CI, confidence interval; HR, adjusted hazard ratio (HGSOC versus LGSOC and other types).

	Univariate				Multiple			
	HR	95% CI		*p*	HR	95% CI		*p*
Age	1.364	0.675	2.753	0.387	*			
FIGO	2.713	1.196	6.153	0.017	1.349	0.362	5.021	0.655
Residual disease	2.084	0.949	4.572	0.067	*			
Peritoneal carcinosis	2.210	1.069	4.568	0.032	0.724	0.225	2.325	0.587
PFI	10.074	3.800	26.706	<0.001	11.341	1.069	120.293	0.044
CTC^combo^ at baseline	0.710	0.189	2.661	0.612	*			
CTC^combo^ at follow-up	6.168	1.326	28.697	0.020	16.588	1.542	178.477	0.020

* Not included in the final multiple Cox regression analysis.

## Data Availability

The data presented in this study are available on request from the corresponding author.

## Supplementary Materials

The following are available online at https://www.mdpi.com/article/10.3390/cancers13112613/s1: Figure S1: association of PPIC gene and protein expression in the primary tumor tissue samples, Table S1: blood samples taken at baseline and follow-up for the detection of CTCs, Table S2: Cox’s proportional hazard regression analysis for survival at baseline, Table S3: Cox’s proportional hazard regression for survival at follow-up.
