# Supplementary material for: The Long-Term Prognostic Significance of Circulating Tumor Cells in Ovarian Cancer—A Study of the OVCAD Consortium
Source: Cancers (Basel). 2021 May 26;13(11):2613. doi: 10.3390/cancers13112613 (PMC8198007; doi:10.3390/cancers13112613)
Supplement: Supplementary file 1 [file cancers-13-02613-s001.zip › cancers-1211678-supplementary.pdf]

cancers

Article

The Long-Term Prognostic Significance of Circulating Tumor Cells in Ovarian Cancer–A Study of the OVCAD Consortium

Eva Obermayr, Angelika Reiner, Burkhard Brandt, Elena Ioana Braicu, Alexander Reinthaller, Liselore Loverix, Nicole Concin, Linn Woelber, Sven Mahner, Jalid Sehouli, Ignace Vergote and Robert Zeillinger

Supplementary Materials:

Citation: Obermayr, E.; Reiner, A.; Brandt, B.; Braicu, E.I.; Reinthaller, A.; Loverix, L.; Concin, N.; Woelber, L.; Mahner, S.; Sehouli, J.; et al. The Long-Term Prognostic Significance of Circulating Tumor Cells in Ovarian Cancer–A Study of the OVCAD Consortium. *Cancers* **2021**, *13*, 2613. https://doi.org/10.3390/cancers13112613

Academic Editor: Noriyoshi Sawabata

Received: 21 April 2021
Accepted: 22 May 2021
Published: 26 May 2021

Publisher's Note: MDPI stays neutral with regard to jurisdictional claims in published maps and institutional affiliations.

Copyright: © 2021 by the authors. Licensee MDPI, Basel, Switzerland. This article is an open access article distributed under the terms and conditions of the Creative Commons Attribution (CC BY) license (http://creativecommons.org/licenses/by/4.0/).

Table S1. Blood samples taken at baseline and follow-up for the detection of CTCs.

**Baseline**	**qPCR**			
	**positive**	negative	not done	total
IF				
positive	5[a]	19[a]	0	24
negative	13[a]	52[b]	1	66
not done	16[a]	94	15[c]	125
Total	34	165	16	215

**Follow-up**	qPCR			
	positive	negative	not done	total
IF				
positive	0	5[a]	0	5
negative	9[a]	43[b]	8	60
not done	4[a]	31	115[c]	150
Total	13	79	123	215

Absolute number of blood samples for the analysis of CTCs by immunfluorescent staining (IF) and/or qPCR. IF was done using antibodies against EpCAM, CK7/18, MUC1, Her2, and EGFR. PCR-positive samples were assigned due to the overexpression of the PPIC gene. [a] positive and [b] negative findings contributing to the combined approach for CTC detection (CTC^{combo}). [c] in these cases no analysis was performed because no blood sample was taken.

Table S2. Cox's proportional hazard regression analysis for survival at baseline.

	univariate				multiple			
**Overall survival**	**HR**	**95% CI**		**p**	**HR**	**95% CI**		**p**
Age	2.119	1.249	3.595	0.005	1.914	1.119	3.274	0.018
FIGO stage	2.398	1.486	3.870	<0.001	1.967	1.211	3.194	0.006
Residual disease	1.910	1.164	3.133	0.010	1.418	0.852	2.360	0.179
Peritoneal carcinosis	1.645	0.948	2.855	0.077	*			
Ascites	2.067	1.145	3.733	0.016	*			
CTC^{combo}	1.869	1.161	3.008	0.010	1.419	0.861	.338	0.170
**Progression-free survival**								
Age	2.289	1.396	3.754	0.001	2.264	1.369	3.744	0.001
FIGO stage	2.162	1.405	3.328	<0.001	1.812	1.190	2.757	0.006
Residual disease	2.535	1.587	4.049	<0.001	1.879	1.152	3.065	0.011
Peritoneal carcinosis	1.855	1.120	3.070	0.016	*			
Ascites	1.407	0.853	2.321	0.181	*			
CTC^{combo}	1.588	1.025	2.460	0.039	1.271	0.811	1.990	0.295

Covariates were patient age (≥ 55 vs <55), FIGO (IIa-IIIb vs. IIIc and IV), residual disease after surgery (yes vs. no), peritoneal carcinosis (yes vs. no), ascites (yes vs. no), and combined CTC results from qPCR and IF (CTC^{combo}-positive vs. CTC^{combo}-negative). CI, confidence interval; HR, hazard ratio adjusted for histological type (HGSOC vs LGSOC and other types); * not included in the multiple Cox regression analysis; n.s. not significant.

Table S3. Cox's proportional hazard regression for survival at follow-up.

	univariate				multiple			
**Overall survival**	**HR**	**95% CI**		**p**	**HR**	**95% CI**		**p**
Age	1.672	0.874	3.196	0.120	*			
FIGO	2.378	1.310	4.315	0.004	1.817	0.955	3.457	0.069
Residual disease	1.361	0.721	2.572	0.342	*			
Peritoneal carcinosis	1.800	0.875	3.703	0.110	*			
Ascites	2.154	0.832	5.581	0.114	*			
CTC^{combo}	3.371	1.704	6.672	<0.001	2.574	1.227	5.398	0.012
**Progression-free survival**								
Age	1.591	0.835	3.032	0.158	*			
FIGO	1.523	0.845	2.744	0.162	*			
Residual disease	1.297	0.673	2.499	0.437	*			
Peritoneal carcinosis	2.119	1.010	4.444	0.047	2.336	1.089	5.010	0.029
Ascites	1.627	0.710	3.730	0.250	*			
CTC^{combo}	3.672	1.817	7.422	<0.001	4.068	1.948	8.498	<0.001

Covariates were patient age (≥ 55 vs <55), FIGO (IIa-IIIb vs. IIIc and IV), residual disease after surgery (yes vs. no), peritoneal carcinomatosis (yes vs. no), ascites (yes vs. no), and combined CTC results from qPCR and IF (CTC^{combo}-positive vs. CTC^{combo}-negative). CI, confidence interval; HR, hazard ratio adjusted for histological type (HGSOC vs LGSOC and other types); * not included in the final multiple Cox regression analysis; n.s. not significant.

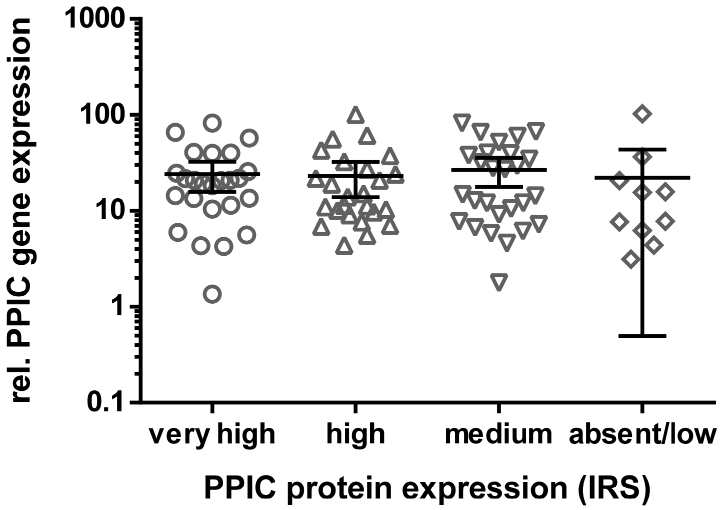

Figure 1. Association of PPIC gene and protein expression in the primary tumor tissue samples. The relative PPIC gene expression was assessed using qPCR and evaluated using the ddCt method with a cell line calibrator sample and a reference gene. PPIC protein expression was assessed by immunohistochemistry in tissue microarrays. The PPIC expression level was scored semi-quantitatively based on staining intensity and percentage of positive tumor cells using the immunoreactive score (IRS).
